# Supplementary material for: High-Level Drug-Resistant Mutations among HIV-1 Subtype A6 and CRF02_AG in Kazakhstan
Source: Viruses. 2023 Jun 21;15(7):1407. doi: 10.3390/v15071407 (PMC10384832; doi:10.3390/v15071407)
Supplement: Supplementary file 1 [file viruses-15-01407-s001.zip › viruses-2412261-supplementary.pdf]

SUPPLEMENTARY MATERIAL

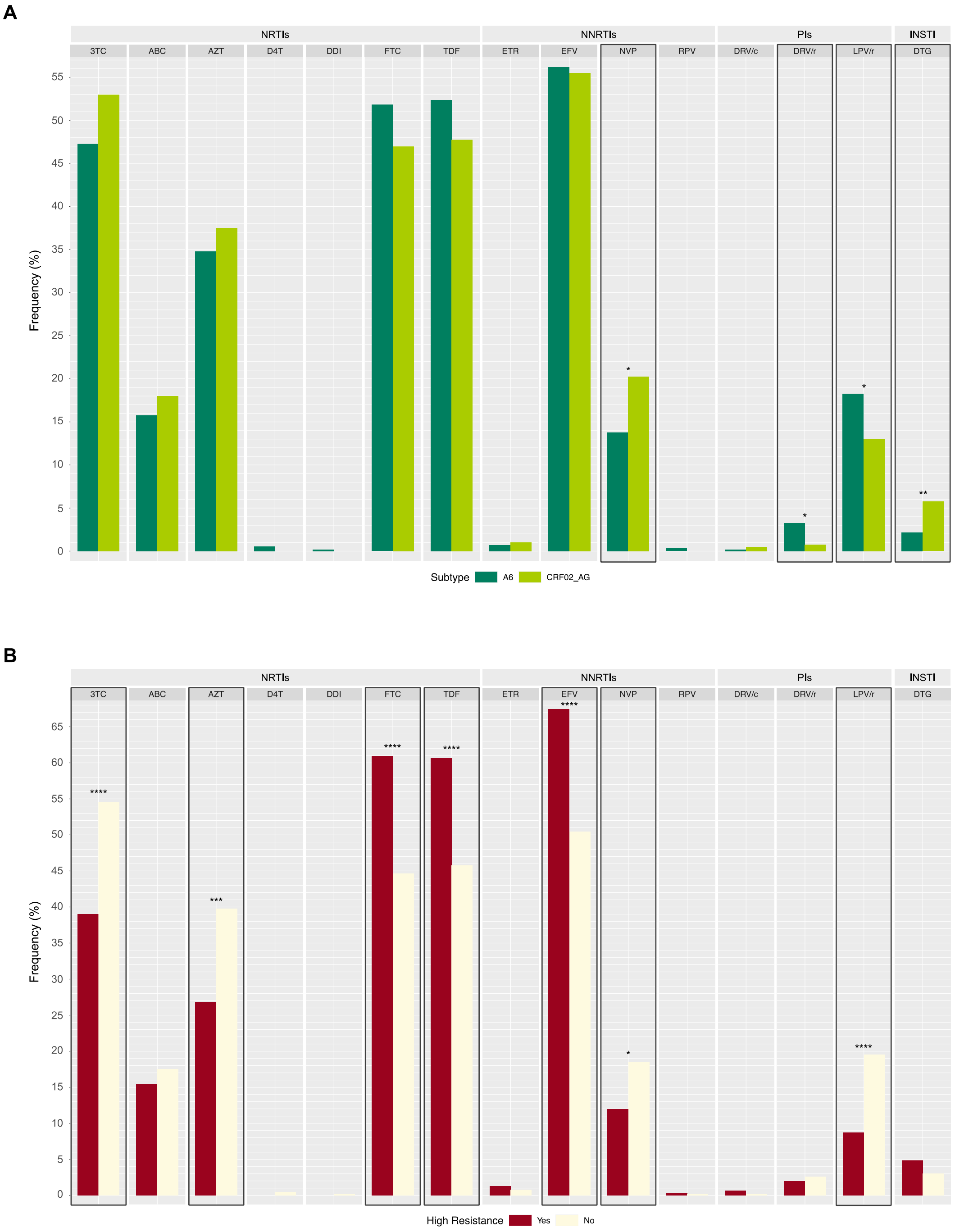

Figure S1. Antiretroviral usage among individuals in the Kazakhstan dataset (*n* = 959, NA = 9), according to HIV subtype (**A**) and presence/absence of high-level drug resistance (**B**). Highlighted boxes indicate significant differences between categories based on χ^2 tests for heterogeneity, with asterisks indicating *p*-value cut-offs (* = *p*-value < 0.05; ** = *p*-value < 0.01; *** = *p*-value < 0.001; **** = *p*-value < 0.0001). Antiretrovirals are grouped by class: nucleoside reverse transcriptase inhibitors (NRTIs), non-nucleoside reverse transcriptase inhibitors (NNRTIs), protease inhibitors (PIs), and one integrase-strand transfer inhibitor (INSTI). 3TC: lamivudine, ABC: abacavir, AZT: zidovudine, D4T: stavudine, DDI: didanosine, FTC: emtricitabine, TDF: tenofovir, ETR: etravirine, EFV: efavirenz, NVP: nevirapine, RPV: rilpivirine, DRV/c: darunavir/cobicistat, DRV/r: darunavir/ritonavir, LPV/r: lopinavir/ritonavir, DTG: dolutegravir.

Table S1. Drug resistant mutations (DRMs) present at over 1% frequency, according to ART generation (*n* = 968).

	1st generation n (%)	*2nd generation n (%)*	*1st and 2nd n (%)*	*NA[1] n (%)*	*Total n (%)*
All sequences	204 (21.1)	464 (47.9)	291 (30.1)	9 (0.9)	968 (100)
NRTI major					
M184V	23 (11.3)	87 (18.8)	38 (13.1)	1 (11.1)	149 (15.4)
K65R	1 (0.5)	36 (7.8)	7 (2.4)	1 (11.1)	45 (4.6)
D67N	2 (1)	10 (2.2)	5 (1.7)	0	17 (1.8)
K70R	2 (1)	10 (2.2)	4 (1.4)	0	16 (1.7)
Y115F	2 (1)	9 (1.9)	4 (1.4)	0	15 (1.5)
M41L	2 (1)	4 (0.9)	8 (2.7)	0	14 (1.4)
K70E	0	9 (1.9)	4 (1.4)	0	13 (1.3)
L74I	1 (0.5)	9 (1.9)	3 (1)	0	13 (1.3)
L74V	2 (1)	3 (0.6)	6 (2.1)	0	11 (1.1)
NRTI accessory					
A62V	43 (21.1)	115 (24.8)	70 (24.1)	5 (55.6)	233 (24.1)
K219E	3 (1.5)	18 (3.9)	2 (0.7)	0	23 (2.4)
NNRTI major					
K103N	28 (13.7)	115 (24.8)	55 (18.9)	1 (11.1)	199 (20.6)
G190S	5 (2.5)	48 (10.3)	8 (2.7)	0	61 (6.3)
Y181C	5 (2.5)	26 (5.6)	11 (3.8)	0	42 (4.3)
P225H	2 (1)	21 (4.5)	8 (2.7)	0	31 (3.2)
G190A	3 (1.5)	6 (1.3)	4 (1.4)	0	13 (1.3)
K238T	3 (1.5)	5 (1.1)	2 (0.7)	0	10 (1)
NNRTI accessory					
K101E	6 (2.9)	30 (6.5)	8 (2.7)	0	44 (4.5)
E138A	11 (5.4)	13 (2.8)	16 (5.5)	0	40 (4.1)
A98G	3 (1.5)	8 (1.7)	5 (1.7)	0	16 (1.7)
V179E	3 (1.5)	10 (2.2)	2 (0.7)	0	15 (1.5)
V108I	6 (2.9)	5 (1.1)	3 (1)	0	14 (1.4)

[1] Sequences from individuals not on ART or with unknown regimens.

ART: antiretroviral therapy, NRTI: nucleoside reverse transcriptase inhibitor, NNRTI: non-nucleoside reverse transcriptase inhibitor.
